# Effect of reliable electricity on health facilities, health information, and child and maternal health services utilization: evidence from rural Gujarat, India

**DOI:** 10.1186/s41043-019-0164-6

**Published:** 2019-02-19

**Authors:** Yvonne Jie Chen, Namrata Chindarkar, Yun Xiao

**Affiliations:** Lee Kuan Yew School of Public Policy, National University of Singapore, 469C Bukit Timah Road, Singapore, 259772 Singapore

**Keywords:** Electrification, Infrastructure, Health systems, Health facilities, Health information, Health services utilization, Difference-in-differences, India

## Abstract

**Background:**

Reliable basic infrastructure, particularly electricity, is a critical enabling factor in improving health systems and consequently achieving the health sustainable development goals (SDGs). Yet, there is no systematic and rigorous study examining the effect of reliable electricity on health systems in a developing country context. In this study, we examine the effect of Jyotigram Yojana (JGY), a rural electrification program providing 24-h electricity to rural non-agricultural users in Gujarat, India, on core components of health systems including health facilities, health information, and health services utilization.

**Methods:**

We match data from the District Level Household and Facility Survey (DLHS-II and DLHS-III) and administrative data from electricity distribution companies on JGY implementation. Matching survey data with administrative data allows us to precisely identify the relevant sample from Gujarat for our data analysis. We then apply a difference-in-differences framework to address potential bias in JGY implementation by comparing the sample from Gujarat (treatment group) with that from Maharashtra (control group). Our key independent variable is a dummy indicating JGY implementation, which operationalizes access to reliable electricity. It takes value 1 if the PHC/eligible woman/child is located or residing in the state of Gujarat and 0 if located or residing in the state of Maharashtra. Our outcome variables cover three core components of health systems—health facilities, health information, and child and maternal health services utilization. Each outcome is a binary variable. We therefore estimate probit models with appropriate control variables.

**Results:**

We find that JGY implementation significantly improved the operational capacity of health facilities, in particular primary health centers (PHCs), by increasing the availability and functionality of a wide range of essential devices and equipment. JGY also significantly increased access to health information through television. Further, JGY increased utilization of health services; in particular, it increased the probability of children receiving critical vaccinations and pregnant women receiving antenatal care. Our results are robust to alternate specifications and analysis using alternate data.

**Conclusion:**

Reliable electricity can be an effective tool in improving core components of health systems. In addition to targeting direct factors within the health systems such as health workforce and health financing, investments in supporting infrastructure are warranted to achieve the health SDGs.

**Electronic supplementary material:**

The online version of this article (10.1186/s41043-019-0164-6) contains supplementary material, which is available to authorized users.

## Background

Much of the policy emphasis towards achieving better health outcomes in developing countries has been on direct factors such as expanding the network of health institutions, training health workforce, and health financing [1]. However, both the United Nations (UN) Sustainable Development Goal (SDG) 3 and the “Global Strategy for Women’s, Children’s and Adolescents’ Health (2016–2030)” recognize that achieving health goals requires an enabling environment that integrates health with other sectors such as basic infrastructure, important among which is electricity [2, 3]. In fact, the World Health Organization (WHO) stresses that electricity is a “critical enabler” of universal access to health care and that without electricity, “many life-saving interventions simply cannot be undertaken” [4].

It is increasingly argued that expanding access to electricity accompanied with reliability, measured using hours of supply and voltage stability, can have much larger welfare effects including impacts on health [4–8]. Previous literature acknowledges availability of electricity as an important determinant of receiving health information and utilization of health services and also as a supply-side prerequisite for health facilities to provide safe and good-quality health services [9–15].

Although there seems to be a clear interaction between electrification and core components of health systems including improvements in primary health facilities and access to health information and health services utilization, there is no systematic and rigorous study linking the two [16]. We address this significant gap in the literature by examining the effects of a unique rural electrification program, Jyotigram Yojana (JGY), launched in 2003 by the state government of Gujarat, India, on health facilities, health information, and health services utilization. JGY used an innovative feeder segregation strategy that rationed the agricultural use of electricity to a pre-scheduled 8 h of uninterrupted, high-quality (three-phase) electricity and guaranteed 24-h high-quality electricity supply to rural non-agricultural users comprising of households, schools, hospitals (including primary health centers (PHCs)), and small commercial users. Additional file 1: Figure S1a and b illustrate the physical infrastructural changes post-JGY [17]. This was a significant improvement over the situation prior to JGY, when rural non-agricultural electricity supply was plagued by power outages and voltage fluctuations [18]. The uniqueness of JGY lies in the fact that electrification expansion under the program was not only about increasing access but also about improving reliability, that is, hours of supply and voltage stability.

A further gap in the literature is empirical evidence from developing countries, where improving both basic infrastructure and health systems are of policy significance. There are at least three methodological challenges in undertaking such analysis. First, large-scale infrastructure projects such as electrification are often planned and therefore suffer from program placement bias. This means that target population or geographical locations chosen earlier might be those where socioeconomic outcomes are low or where there might be most political interest [7, 19, 20]. Second, in developing countries, electrification (or infrastructure more generally) expansion and improvements to health systems may happen simultaneously as both are priority sectors. These two challenges make it difficult to attribute any changes in health outcomes solely to electrification expansion. A third challenge has to do with access to administrative data on the implementation of infrastructure projects. In the absence of such data, researchers often rely on proxies such as constructing the variable for exposure to policy from other secondary sources [21]. However, such proxies can under- or overestimate on-the-ground realities such as the speed and intensity of project implementation.

The methodology adopted in this paper attempts to overcome the empirical challenges highlighted above. We use a novel approach of matching population-based survey data representative at the district level and administrative data on JGY implementation to examine the effect of reliable electricity on core components of health systems. To our knowledge, this is the first study to provide rigorous empirical evidence on the effects of large-scale infrastructure improvements on health systems in a developing country. Our analysis shows that JGY implementation significantly improved the operational capacity of health facilities, in particular primary health centers (PHCs), by increasing the availability and functionality of a wide range of essential devices and equipment that require reliable electricity. JGY also significantly increased access to health information through television. Further, JGY increased utilization of health services; in particular, it increased the probability of children receiving critical vaccinations and pregnant women receiving antenatal care. Our results are robust to alternate specifications and analysis using alternate data.

## Methods

Data for this study primarily come from two sources—JGY implementation data obtained from the electricity distribution companies and repeated cross-sections from two rounds of survey data from the District Level Household and Facility Survey (DLHS-II (2002–2004) and DLHS-III (2007–2008)) [22, 23]. JGY implementation data were obtained through administrative records provided by the four regional distribution companies in the state of Gujarat. These companies together cover all districts (sub-region of a state) of Gujarat. The administrative data record the exact timing (year-month) when feeder segregation under JGY was started and completed in each village (sub-region of a district) in Gujarat. As DLHS does not provide village names, we match JGY data with DLHS data at the district level. More specifically, we aggregate the village-level data to identify JGY implementation in each district, where “implementation” refers to completion of feeder segregation in 100% of the villages in a given district. We then match the information on JGY implementation with relevant information from the survey to identify the samples for our data analysis.

DLHS follows a two-stage stratified sampling method in rural areas and three-stage stratified sampling method in urban areas. It covers all census districts and is therefore representative at the district level. The main survey instrument for DLHS comprises of three sets of questionnaires: household, ever married women, and unmarried women. It also includes health facility questionnaires. For examining health facilities outcomes, we draw on data from the primary health center (PHC) questionnaire, and for the health information and health services utilization outcomes, we use the ever-married women questionnaires from DLHS-II and DLHS-III.

As previously stated, large-scale infrastructure projects suffer from program placement bias [7, 19, 20]. Our administrative data also suggest that districts were not chosen randomly for JGY implementation (see Additional file 1: Figure S2). We therefore cannot rule out the possibility that the districts chosen earlier for JGY and the speed of implementation in each district (from start date to completion date) are systematically correlated with district-level socioeconomic and unobserved factors. If unobserved district characteristics are correlated with both the JGY implementation and outcome variables, our analysis would be biased.

**Fig. 1:**
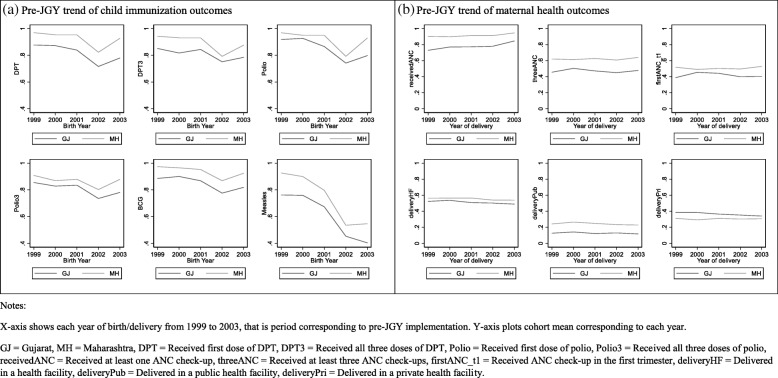
Pre-JGY trends of child immunization and maternal health outcomes using DLHS-II data. **a** Pre-JGY trend of child immunization outcomes. **b** Pre-JGY trend of maternal health outcomes. *X*-axis shows each year of birth/delivery from 1999 to 2003, that is period corresponding to pre-JGY implementation. *Y*-axis plots cohort mean corresponding to each year. GJ, Gujarat; MH, Maharashtra; DPT, received first dose of DPT; DPT3, received all three doses of DPT; Polio, received first dose of polio; Polio3, received all three doses of polio; receivedANC, received at least one ANC check-up; threeANC, received at least three ANC check-ups; firstANC_t1, received ANC check-up in the first trimester; deliveryHF, delivered in a health facility; deliveryPub, delivered in a public health facility; deliveryPri, delivered in a private health facility

To address this, we use a difference-in-differences framework where PHCs/eligible women/children in Gujarat form the treatment group and those in the neighboring state of Maharashtra form the control group. No electrification expansion program was implemented in Maharashtra prior to or simultaneously with JGY. We then compare PHC/eligible women/children outcomes in Gujarat before and after the implementation of JGY with that of the neighboring state of Maharashtra. The intuition underlying the difference-in-differences framework is that at the baseline, that is, prior to any intervention, the difference in the outcome between the treatment and control groups follows a parallel trend. This essentially means that all other factors, besides the intervention, that the two groups are exposed to are similar. Therefore, any “shift” in the trend after the intervention is introduced can be attributed to the intervention. In our context, the assumption is that districts in Gujarat and Maharashtra followed a parallel trend with regards to the outcomes prior to JGY implementation. We use descriptive statistics and trend analysis of relevant indicators prior to JGY implementation to establish that the two states were on average similar. Pre-JGY trends are plotted for child and maternal health outcomes using yearly cohorts of children and eligible women from DLHS-II who were born or gave birth between 1999 and 2003, which is the period corresponding to pre-JGY implementation, from Gujarat and Maharashtra. From the descriptive statistics in Table 1 and pre-JGY trends in Fig. 1a and b, we conclude that the two states are comparable at the baseline. We then estimate regression models and interpret the post-JGY change in outcomes in Gujarat as that attributable to JGY implementation.

**Table 1:** Baseline characteristics (pre-JGY) of Gujarat and Maharashtra

	Gujarat	Maharashtra	Reference period	Source
Rural population as a percentage of total population	63	58	2001	Census 2001
Percentage of electrified rural households	72	65	2001	Census 2001
Average rural population covered per primary health center (PHC)	31,666	31,523	As of Mar. 30, 2001	Institute of Applied Manpower Research Yearbook
Population served per government hospital bed	1564	1325	Gujarat: as of Jan. 1, 2004 Maharashtra: as of Sept. 1, 2004	Ministry of Health and Family Welfare
Percentage of infants immunized under 20-point program	84	89	As of Sept. 1, 2003	Ministry of Statistics and Programme Implementation
Percentage of pregnant women receiving at least one antenatal check-up	88	93	2002–2004	District Level Household Survey (DLHS)–II

All statistics were sourced from https://www.indiastat.com/ [39]. The column “Source” refers to original data source as per https://www.indiastat.com/

The full list of outcome variables for health facilities, health information, and child and maternal health services utilization is provided in Table 2. We include relevant outcomes that are consistently available in both DLHS-II and DLHS-III questionnaires. Each outcome is a binary variable. We therefore estimate probit models using the statistical software Stata 14 [24]. Our key independent variable is a dummy indicating JGY implementation, which operationalizes access to reliable electricity. It takes value 1 if the PHC/eligible woman/child is located or residing in the state of Gujarat and 0 if located or residing in the state of Maharashtra. Matching survey data with administrative data allows us to precisely identify the treated sample from Gujarat. Based on the timing of JGY implementation, the sample from Gujarat varies for the three sets of outcomes. Details of the samples used in the data analysis are also provided in Table 2. Additional demographic and socioeconomic controls included in the regressions for the three sets of outcomes vary as the units of analysis are different. Table 2 lists the full set of control variables.

**Table 2:** Details of outcome variables, sample, and control variables included in the analysis

Outcome variables	Variable description	Unit of analysis	Sample	Control variables
I. Health facility (PHC)				
1. Availability of electricity 2. Need for a generator 3. Functioning deep freezer 4. Functioning ice-lined refrigerator 5. Functioning cold box 6. Functioning vaccine carrier 7. Functioning delivery room 8. Functioning operating table 9. Functioning delivery table 10. Functioning examination table	Dummy variable taking value 1 if the essential device/equipment is available and functional, 0 otherwise.	Primary health center (PHC)	All PHCs interviewed in DLHS-II (pre-JGY) and DLHS-III (post-JGY) in Gujarat and Maharashtra.	Type of building (kaccha, semi-pucca, pucca), access to piped water, access to toilet, district fixed effects, and interview year fixed effects. In addition, district-level pre-JGY health status (proxied using under-five mortality rate in 2001) interacted with interview year dummies is included to control for unobserved differences across districts that may violate the parallel trend assumption.
II. Health information				
1. Ever heard of HIV/AIDS If heard, heard from: 2. TV 3. Any other source	Dummy variable taking value 1 if response is “Yes,” 0 otherwise.	Eligible woman (women aged 18–45 that ever gave birth)	Timing of JGY implementation is matched with timing of the household interviews in Gujarat. Sample includes eligible women interviewed in DLHS-II (pre-JGY) and DLHS-III (post-JGY) from Gujarat and Maharashtra but excluding those residing in districts in Gujarat where JGY was already implemented in 2003–2004.	Age of the eligible woman, age squared, years of education of eligible woman, husband’s years of education, age of the household head, gender of the household head, religion and social group of the eligible woman, household standard of living index, distance to nearest health facility, district fixed effects, and interview year fixed effects. In addition, district-level pre-JGY health status (proxied using under-five mortality rate in 2001) interacted with interview year dummies is included to control for unobserved differences across districts that may violate the parallel trend assumption.
III. Health services utilization				
A. Child immunization services 1. Received first DPT dose 2. Received all three DPT doses 3. Received first polio dose 4. Received all three polio doses 5. Received BCG vaccine 6. Received measles vaccine	Dummy variable taking value 1 if the child receives the specific immunization, 0 otherwise.	Child (children between 0 and 36 months old at the time of survey)	Timing of JGY implementation is matched with timing of birth of a child such that only those children in Gujarat born before JGY implementation in DLHS-II and those born after JGY implementation in DLHS-III are retained in the sample. All children from Maharashtra in DLHS-II and DLHS-III are included.	Child’s age in months, mother’s age at birth, mother’s years of education, father’s years of education, birth order, dummy for multiple birth, age of the household head, gender of the household head, religion and social group of the child, household size, household standard of living index, distance to nearest health facility, district fixed effects, and birth year fixed effects. In addition, district-level pre-JGY health status (proxied using under-five mortality rate in 2001) interacted with birth year dummies is included to control for unobserved differences across districts that may violate the parallel trend assumption.
B. Maternal health services (antenatal care and institutional delivery) 1. Received at least one ANC check-up 2. Received at least three ANC check-ups 3. Received ANC check-up in the first trimester 4. Delivery in a health facility 5. Delivery in a public health facility 6. Delivery in a private health facility	Dummy variable taking value 1 if the pregnant woman receives the specific ANC service, 0 otherwise.	Eligible woman (women who gave live or still birth to a child in the past 2 years)	Timing of JGY implementation is matched with the timing of delivery such that only those eligible women in Gujarat who gave birth prior to JGY implementation in DLHS-II and those in DLHS-III who gave birth after JGY implementation are retained in the sample. All eligible women from Maharashtra in DLHS-II and DLHS-III are included.	Eligible woman’s age at delivery, age squared, total number of births, total number of pregnancies, years of education of eligible woman, husband’s years of education, age of the household head, gender of the household head, religion and social group of the eligible woman, household size, household standard of living index, distance to nearest health facility, district fixed effects, and delivery year fixed effects. In addition, district-level pre-JGY health status (proxied using under-five mortality rate in 2001) interacted with delivery year dummies is included to control for unobserved differences across districts that may violate the parallel trend assumption.

Our probit regression model is as follows:

1 $$ {y}_{ist}={\alpha}_0+{\alpha}_1{T}_s+{\alpha}_2{P}_t+{\alpha}_3{T}_s\times {P}_t+{\delta}_d+{\varphi}_v\times {X}_d+{\varepsilon}_{ist} $$

where *y*_*ist*_ is the binary outcome variable for PHC/eligible woman/child *i* in state *s* and survey round *t*. *T*_*s*_ takes value 1 if the PHC/eligible woman/child is in Gujarat and 0 if it is in Maharashtra. *P*_*t*_ takes value 1 if a PHC/eligible woman/child was interviewed/gave birth/born post-JGY implementation and 0 otherwise. *ε*_*ist*_ is the random error. Standard errors are clustered at the district level.

Other electrification and health programs that were implemented contemporaneously with JGY might confound our outcome variables or contaminate the control group from Maharashtra. These programs include the Rajiv Gandhi Grameen Vidyutikaran Yojana—a national-level program introduced in 2005 and aimed at providing free electricity to below poverty line (BPL) households; Janani Suraksha Yojana—a national-level program introduced in 2005 and aimed at promoting institutional delivery; and Chiranjeevi Yojana— a state-level program introduced in 2005 in Gujarat that follows a public-private partnership model to promote institutional delivery. Not controlling for the effects of these programs might bias our estimates upwards, that is, we may overestimate the effect of JGY on our outcomes. To address this, we include district and year fixed effects in our regression models. Year fixed effects control for unobserved factors that are district-invariant, that is, unobserved factors common to all districts in a given year such as other national- and state-level electrification and health policies. District fixed effects control for unobserved factors that are time-invariant, that is, they do not change for a given district over time. These might include geographical and administrative characteristics of a district that affect program implementation. *δ*_*d*_ and *φ*_*v*_ represent district and interview/delivery/birth year fixed effects, respectively.

Inclusion of district and year fixed effects does not account for across-district differences in unobserved factors prior to JGY implementation, which may violate the parallel trend assumption. For instance, if some districts trail behind on health or development outcomes, they may receive priority treatment when implementing policies. We therefore include the interaction term *X*_*d*_ × *φ*_*v*_, which is the district-level pre-JGY health status interacted with interview/delivery/birth year fixed effects. Here, the district-level pre-JGY health status is a proxy for broader conditions pertaining to health and development in a district that existed prior to JGY implementation, which may bias not only the JGY implementation across districts but also the implementation of other electrification and health programs discussed above. The interaction term therefore explicitly controls for any sharp deviations in district trends resulting from pre-JGY unobserved differences across districts that may violate the parallel trend assumption [25].

The difference-in-differences coefficient of interest is *α*_3_, which estimates the impact of reliable electricity on the outcomes post-JGY implementation in Gujarat. The coefficient on *α*_1_ is the state average effect across all districts in Gujarat, while the coefficient on *α*_2_ is the district average over the post-JGY period. Theoretically, when district and interview/delivery/birth year fixed effects are included, *α*_1_ and *α*_2_ are absorbed.

## Results

Table 3 summarizes all the outcome and control variables included in our analysis for the samples from Gujarat (treatment group) and Maharashtra (control group). We report the summary statistics in four separate panels—health facilities, health information, child immunization services, and maternal health services (antenatal care and institutional delivery). Only samples used for regression analyses are included in the summary statistics. Overall, the control variables show similarities between Gujarat and Maharashtra over many dimensions, including age of household head, gender of household head, household size, and household standard of living index.

**Table 3:** Summary statistics

Variables	Gujarat			Maharashtra		
	*N*	Mean	SD	*N*	Mean	SD
Panel A. Health facility sample (*N* = 1816)						
Outcome variables						
Electricity	638	0.975	0.156	1178	0.960	0.196
Generator	638	0.592	0.492	1178	0.772	0.419
Ice-lined refrigerator	638	0.934	0.248	1178	0.964	0.186
Deep freezer	638	0.923	0.266	1178	0.951	0.216
Cold box	638	0.950	0.218	1178	0.975	0.155
Vaccine carrier	638	0.969	0.174	1178	0.992	0.0871
Delivery room	638	0.644	0.479	1178	0.776	0.417
Operating table	638	0.125	0.331	1178	0.735	0.441
Delivery table	638	0.909	0.288	1178	0.941	0.237
Examination table	638	0.933	0.251	1178	0.944	0.230
Control variables						
JGY program	638	0.442	0.497	1178	0	0
Gujarat	638	1	0	1178	0	0
Interview in 2007–2008	638	0.442	0.497	1178	0.425	0.495
Kaccha building	638	0.009	0.097	1178	0.032	0.177
Semi-pucca building	638	0.110	0.313	1178	0.228	0.419
Pucca building	638	0.881	0.324	1178	0.740	0.439
Piped water	638	0.580	0.494	1178	0.492	0.500
Toilet	638	0.929	0.256	1178	0.871	0.335
Panel B. Health information sample (*N* = 34,651)						
Outcome variables						
Ever heard of HIV/AIDS	13,321	0.325	0.468	21,326	0.649	0.477
Heard from TV	13,321	0.210	0.407	21,326	0.392	0.488
Heard from radio	13,321	0.0533	0.225	21,326	0.121	0.326
Heard from newspaper	13,321	0.0882	0.284	21,326	0.122	0.327
Heard from health workers	13,321	0.0329	0.178	21,326	0.0419	0.200
Control variables						
JGY program	13,323	0.608	0.488	21,328	0	0
Gujarat	13,323	1	0	21,328	0	0
Interview in 2007–2008	13,323	0.608	0.488	21,328	0.591	0.492
Mother’s age in year	13,323	30.96	8.116	21,328	30.24	8.229
Mother’s age squared	13,323	1024	520.5	21,328	982.1	518.0
Standard of living index	13,323	1.754	0.770	21,328	1.662	0.729
Respondent’s years of schooling	13,323	3.470	4.267	21,328	4.660	4.390
Husband’s years of schooling	13,323	6.113	6.169	21,328	6.834	6.205
Age of the head	13,323	43.79	12.42	21,321	46.30	13.67
Female head	13,323	0.044	0.205	21,321	0.051	0.220
Distance to nearest health facility (km)	13,288	3.856	5.316	20,869	4.240	5.693
Panel C. Child immunization sample (*N* = 9580)						
Outcome variables						
Received DPT first dose	3371	0.829	0.376	5659	0.865	0.342
Received DPT all three doses	3006	0.691	0.462	5313	0.706	0.456
Received Polio first dose	3393	0.864	0.343	5693	0.891	0.312
Received Polio all three doses	3044	0.721	0.448	5292	0.742	0.437
Received BCG	3387	0.884	0.320	5687	0.919	0.273
Received Measles	3367	0.502	0.500	5655	0.488	0.500
Control variables						
JGY program	3608	0.472	0.499	5972	0	0
Gujarat	3608	1	0	5972	0	0
Birth in 2007–2008	3608	0.472	0.499	5972	0.567	0.496
Age in months	3608	12.99	7.400	5972	11.97	7.170
Mother’s age at birth	3608	23.74	4.485	5972	22.14	4.198
Standard of living index	3608	5.831	8.110	5972	6.266	7.880
Mother’s years of schooling	3608	4.263	4.467	5972	5.738	4.458
Father’s years of schooling	3608	6.459	4.584	5972	7.385	4.751
Birth order	3608	2.363	1.476	5972	2.199	1.377
Multiple birth	3608	0.987	0.112	5972	0.991	0.0964
Household size	3608	7.174	2.875	5972	7.251	3.110
Age of the head	3608	44.02	14.40	5972	46.21	15.85
Female head	3608	0.0432	0.203	5972	0.0429	0.203
Distance to nearest health facility (km)	3600	2.038	4.365	5903	2.612	4.798
Panel D. Maternal health sample (*N* = 9543)						
Outcome variables						
At least one check-up	3578	0.767	0.423	5965	0.905	0.293
At least three check-ups	3557	0.524	0.499	5913	0.672	0.469
Check-up in the first trimester	3545	0.460	0.498	5921	0.536	0.499
Delivery in a health facility	3578	0.482	0.500	5965	0.509	0.500
Delivery in a public facility	3578	0.177	0.382	5965	0.243	0.429
Delivery in a private facility	3578	0.285	0.452	5965	0.260	0.439
Control variables						
JGY program	3578	0.484	0.500	5965	0	0
Gujarat	3578	1	0	5965	0	0
Delivery in 2007–2008	3578	0.484	0.500	5965	0.582	0.493
Mother’s age in year	3578	24.06	4.539	5965	22.41	4.228
Mother’s age squared	3578	599.6	237.8	5965	520.0	213.3
Standard of living index	3578	5.904	8.085	5965	6.392	7.882
Mother’s years of schooling	3578	4.277	4.474	5965	5.734	4.457
Father’s years of schooling	3578	6.674	6.486	5965	7.563	6.351
Total number of births	3578	2.452	1.508	5965	2.214	1.382
Household size	3578	7.075	2.854	5965	7.188	3.121
Age of the head	3578	44.03	14.37	5965	46.19	15.85
Female head	3578	0.0442	0.205	5965	0.0438	0.205
Distance to nearest health facility (km)	3569	2.090	4.420	5893	2.701	4.868

Table reports number of observations, mean values, and sample standard deviation for observations included in regression analyses. Refer to Table 2 for detailed variable description and units of analysis

We report marginal effect at means from the probit regressions, which can be interpreted as the probability change in the outcome variable taking value 1 post-JGY implementation in Gujarat. We first investigate the effect of JGY implementation (reliable electricity) on health facilities, that is, PHCs. Table 4, columns (1)–(10), reports the effect of JGY implementation on ten outcomes that capture operational capacity of PHCs. We define operational capacity as the availability and functionality of essential devices and equipment that directly or indirectly require reliable electricity. We find that post-JGY implementation in Gujarat the probability of a PHC reporting availability of electricity increased significantly by 12.7%. The need for a generator decreased by 21.5%. The probability of a functioning deep freezer, ice-lined refrigerator, cold box, and vaccine carrier increased significantly by 6.5%, 5.2%, 5.8%, and 6.6%, respectively. Further, the probability of a functioning operating table and delivery table also increased by 10.3% and 6%, respectively.

**Table 4:** Difference-in-differences estimates of the effect of JGY implementation on health facilities (PHCs)

Variables	(1)	(2)	(3)	(4)	(5)	(6)	(7)	(8)	(9)	(10)
	Electricity	Generator	Deep freezer	Ice-lined refrigerator	Cold box	Vaccine carrier	Delivery room	Operating table	Delivery table	Examination table
Post*Gujarat (JGY program effect)	0.127*** (0.050–0.204)	− 0.215*** (− 0.373 to − 0.056)	0.065** (0.012–0.118)	0.052** (0.003–0.101)	0.058** (0.003–0.114)	0.066** (0.010–0.123)	0.060 (− 0.012–0.131)	0.103** (0.010–0.197)	0.060** (0.003–0.117)	0.011 (− 0.046–0.068)
Gujarat	− 0.045*** (− 0.061 to − 0.029)	0.326*** (0.265–0.388)	− 0.082*** (− 0.099 to − 0.065)	− 0.023 (− 0.054–0.008)	− 0.077*** (− 0.093 to − 0.062)	− 0.326*** (− 0.376 to − 0.276)	− 0.005 (− 0.024–0.014)	− 0.424*** (− 0.465 to − 0.383)	− 0.108*** (− 0.128 to − 0.088)	− 0.088*** (− 0.109 to − 0.067)
Post (interview in 2007–2008)	0.228** (0.050–0.406)	0.241* (− 0.044–.526)	− 0.023 (− 0.127–0.081)	− 0.163*** (− 0.280 to − 0.047)	0.029 (− 0.042–0.099)	0.008 (− 0.112–0.127)	0.068 (− 0.056–0.192)	0.007 (− 0.119–0.133)	− 0.001 (− 0.122–0.120)	− 0.028 (− 0.153–0.097)
Semi-pucca building	0.027 (− 0.011–0.066)	0.005 (− 0.126–0.136)	0.041 (− 0.017–0.100)	− 0.001 (− 0.072–0.071)	0.035 (− 0.030–0.100)	− 0.285*** (− 0.322 to − 0.247)	0.196*** (0.060–0.332)	0.079 (− 0.025–0.184)	0.023 (− 0.038–0.084)	0.011 (− 0.066–0.088)
Pucca building	0.063*** (0.025–0.102)	0.029 (− 0.105–0.163)	0.063*** (0.021–0.106)	0.039 (− 0.025–0.104)	0.048* (− 0.008–0.104)	− 0.272*** (− 0.291 to − 0.253)	0.247*** (0.125–0.370)	0.172*** (0.075–0.270)	0.054* (− 0.003–0.111)	0.019 (− 0.063–0.101)
Piped water	0.033*** (0.012–0.055)	0.017 (− 0.036–0.070)	0.026* (− 0.001–0.054)	0.011 (− 0.022–0.043)	0.008 (− 0.018–0.035)	0.007 (− 0.026–0.041)	0.047** (0.009–0.085)	0.018 (− 0.012–0.049)	0.025** (0.004–0.046)	0.010 (− 0.018–0.039)
Toilet	0.099*** (0.077–0.121)	0.195*** (0.127–0.263)	0.052*** (0.024–0.079)	0.047** (0.008–0.087)	0.049*** (0.012–0.085)	0.037*** (0.011–0.064)	0.434*** (0.382–0.485)	0.412*** (0.352–0.472)	0.126*** (0.103–0.149)	0.081*** (0.049–0.112)
Observations	1183	1803	1468	1519	1384	793	1816	1756	1739	1610

Reported coefficients are probit marginal effects. Standard errors clustered at the district level. 95% confidence intervals in parentheses. Regressions include district fixed effects, interview year fixed effects, and district-level pre-JGY health status interacted with interview year dummies. ***p < 0.01, **p < 0.05, *p < 0.1

Table 5 reports the effect of JGY implementation on receiving health information on HIV/AIDS. We find that JGY implementation does not have any effect on general awareness about HIV/AIDS. However, among eligible women who reported being aware of these health conditions, the probability of receiving information via television increased significantly by 5.7% post-JGY. No increase in the probability of receiving health information via any other source was reported. Unfortunately, DLHS-II and DLHS-III do not contain variables on information gained via the internet or smart phones.

**Table 5:** Effect of JGY implementation on health information

Variables	(1)	(2)	(3)
	Ever heard of HIV/AIDS	Heard from TV	Any other source
Post*Gujarat (JGY program effect)	0.033 (− 0.040–0.105)	0.057** (0.005–0.110)	0.001 (− 0.072–0.074)
Gujarat	− 0.269*** (− 0.426 to − 0.113)	0.108 (− 0.026–0.241)	− 0.153** (− 0.306 to − 0.001)
Post (interview in 2007–2008)	− 0.152*** (− 0.196 to − 0.108)	− 0.266*** (− 0.294 to − 0.237)	0.136*** (0.092–0.180)
Age	0.016*** (0.012–0.020)	0.001 (− 0.006–0.007)	0.013*** (0.006–0.019)
Age squared	− 0.000*** (− 0.000 to − 0.000)	− 0.000 (− 0.000–0.000)	− 0.000*** (− 0.000 to − 0.000)
Years of schooling	0.036*** (0.035–0.038)	0.022*** (0.019–0.024)	0.007*** (0.004–0.009)
Husband’s years of schooling	0.003*** (0.002–0.004)	0.004*** (0.002–0.006)	− 0.001 (− 0.002–0.000)
Age of the head	− 0.000** (− 0.001 to − 0.000)	− 0.000 (− 0.001–0.000)	0.000 (− 0.000–0.000)
Female head	0.011 (− 0.008–0.030)	0.014 (− 0.026–0.053)	0.017 (− 0.004–0.039)
Standard of living index	0.081*** (0.072–0.090)	0.148*** (0.135–0.161)	− 0.022*** (− 0.034 to − 0.009)
Distance to nearest health facility	− 0.001 (− 0.003–0.000)	− 0.000 (− 0.002–0.001)	0.001 (− 0.001–0.002)
Observations	34,430	18,093	18,093

Reported coefficients are probit marginal effects. Regressions include religion and social group dummies, district fixed effects and interview year fixed effects, and district-level pre-JGY health status interacted with interview year dummies. Standard errors clustered at the district level. 95% confidence intervals in parentheses. ***p < 0.01, **p < 0.05, *p < 0.1

Finally, we examine health services utilization, in particular, child immunization and maternal health (antenatal care and institutional delivery) services. Results are reported in Tables 6 and 7. For child immunization, we look at the effect of JGY on four key vaccines included in the immunization schedule recommended by the Indian Academy of Pediatrics [26]. Table 6 shows that the probability of receiving the first dose of DPT vaccine, first dose of polio vaccine, BCG vaccine, and measles vaccine increased significantly post-JGY implementation in Gujarat. We also find a marginal increase (*p* < 0.10) of 3.6% in the probability of receiving all three doses of polio vaccine. Table 7 reports the effect of JGY on antenatal check-ups and institutional delivery. We find that in the post-JGY implementation in Gujarat, the probability of receiving a check-up in the first trimester increased significantly by 9.5%. However, we do not find any effects of JGY on institutional delivery or delivery in a public facility.

**Table 6:** Effect of JGY implementation on health services utilization—child immunization

Variables	(1)	(2)	(3)	(4)	(5)	(6)
	DPT		Polio		BCG	Measles
	First dose	All doses	First dose	All doses		
Post*Gujarat (JGY program effect)	0.074*** (0.030–0.118)	0.035 (− 0.015–0.085)	0.038** (0.000–0.076)	0.036* (− 0.005–0.077)	0.064*** (0.027–0.102)	0.122*** (0.057–0.187)
Gujarat	− 0.025 (− 0.076–0.026)	− 0.140*** (− 0.206 to − 0.073)	0.025 (− 0.022–0.073)	− 0.082** (− 0.149 to − 0.016)	− 0.056** (− 0.109 to − 0.004)	0.070 (− 0.037–0.177)
Post (birth in 2007–2008)	− 0.075 (− 0.204–0.053)	− 0.317*** (− 0.495 to − 0.140)	0.064 (− 0.044–0.172)	− 0.231*** (− 0.398 to − 0.064)	0.125* (− 0.016–0.265)	− 0.166 (− 0.430–0.099)
Age in months	0.015*** (0.013–0.017)	0.024*** (0.022–0.027)	0.013*** (0.011–0.015)	0.025*** (0.022–0.027)	0.009*** (0.007–0.011)	0.038*** (0.035–0.040)
Mother’s age at birth	0.003*** (0.001–0.005)	0.006*** (0.003–0.008)	0.002** (0.000–0.004)	0.005*** (0.002–0.007)	0.004*** (0.001–0.006)	0.004*** (0.002–0.007)
Mother’s years of schooling	0.006*** (0.004–0.008)	0.005*** (0.002–0.008)	0.004*** (0.001–0.006)	0.004*** (0.002–0.007)	0.007*** (0.005–0.009)	0.004*** (0.001–0.008)
Father’s years of schooling	0.004*** (0.002–0.006)	0.003** (0.001–0.005)	0.003*** (0.001–0.005)	0.004*** (0.002–0.006)	0.004*** (0.002–0.006)	0.005*** (0.003–0.007)
Birth order	− 0.013*** (− 0.019 to − 0.006)	− 0.009** (− 0.016 to − 0.002)	− 0.010*** (− 0.016 to − 0.005)	− 0.003 (− 0.011–0.005)	− 0.008*** (− 0.015 to − 0.002)	− 0.016*** (− 0.024 to − 0.008)
Multiple birth	0.030 (− 0.049–0.109)	− 0.054 (− 0.166–0.058)	0.042 (− 0.031–0.115)	0.002 (− 0.100–0.103)	0.009 (− 0.049–0.066)	− 0.030 (− 0.152–0.092)
Age of the head	0.000 (− 0.000–0.001)	0.000 (− 0.001–0.001)	− 0.000 (− 0.001–0.000)	0.000 (− 0.001–0.001)	− 0.000 (− 0.001–0.000)	− 0.000 (− 0.001–0.000)
Female head	0.014 (− 0.017–0.044)	− 0.016 (− 0.053–0.022)	0.005 (− 0.023–0.033)	− 0.004 (− 0.038–0.030)	0.001 (− 0.033–0.036)	0.003 (− 0.045–0.050)
Household size	− 0.002 (− 0.005–0.001)	− 0.001 (− 0.004–0.002)	− 0.000 (− 0.003–0.002)	− 0.002 (− 0.005–0.001)	− 0.001 (− 0.003–0.001)	− 0.001 (− 0.004–0.003)
Standard of living index	− 0.001 (− 0.003–0.001)	0.002*** (0.001–0.004)	− 0.003*** (−0.004 to − 0.001)	0.001 (− 0.000–0.003)	− 0.001 (− 0.002–0.001)	− 0.002** (− 0.004 to − 0.000)
Distance to nearest health facility	0.000 (− 0.002–0.002)	−0.001 (− 0.003–0.001)	0.000 (− 0.002–0.002)	0.001 (− 0.001–0.003)	− 0.002** (−0.004 to − 0.000)	0.002 (− 0.001–0.004)
Observations	8988	8285	9044	8302	8792	8980

Reported coefficients are probit marginal effects. Regressions include religion and social group dummies, district fixed effects, birth year fixed effects, and district-level pre-JGY health status interacted with birth year dummies. Standard errors clustered at the district level. 95% confidence intervals in parentheses. ***p < 0.01, **p < 0.05, *p < 0.1

**Table 7:** Effect of JGY implementation on health services utilization—maternal health

VARIABLES	(1)	(2)	(3)	(4)	(5)	(6)
	At least one ANC check-up	At least three ANC check-ups	ANC check-up in the first trimester	Delivery in health facility	Delivery in public facility	Delivery in private facility
Post*Gujarat (JGY program effect)	− 0.005 (− 0.050–0.040)	0.038 (− 0.022–0.098)	0.095*** (0.023–0.166)	− 0.009 (− 0.058–0.039)	0.042 (− 0.009–0.094)	− 0.026 (− 0.077–0.025)
Gujarat	− 0.225*** (−0.269 to − 0.180)	− 0.225*** (− 0.298 to − 0.152)	− 0.164*** (− 0.236 to − 0.092)	0.080*** (0.021–0.139)	0.133*** (0.080–0.185)	− 0.036 (− 0.098–0.027)
Post (delivery in 2007–2008)	0.027 (− 0.097–0.150)	− 0.020 (− 0.207–0.167)	0.151* (− 0.028–0.329)	− 0.023 (− 0.197–0.150)	0.001 (− 0.146–0.148)	−0.073 (− 0.231–0.085)
Age at delivery	0.012*** (0.005–0.019)	0.023*** (0.009–0.036)	0.013* (− 0.001–0.028)	0.008 (− 0.009–0.025)	0.006 (− 0.008–0.021)	0.001 (− 0.016–0.017)
Age squared	− 0.000** (− 0.000 to − 0.000)	− 0.000*** (− 0.001 to − 0.000)	− 0.000 (− 0.000–0.000)	0.000 (− 0.000–0.000)	− 0.000 (− 0.000–0.000)	0.000 (− 0.000–0.000)
Total number of births	− 0.018*** (− 0.025 to − 0.011)	− 0.034*** (− 0.044 to − 0.024)	− 0.035*** (− 0.046 to − 0.025)	− 0.070*** (− 0.079 to − 0.062)	− 0.022*** (− 0.031 to − 0.013)	− 0.052*** (− 0.060 to − 0.044)
Years of schooling	0.015*** (0.012–0.017)	0.021*** (0.018–0.024)	0.018*** (0.015–0.021)	0.022*** (0.018–0.025)	0.006*** (0.003–0.009)	0.016*** (0.013–0.018)
Husband’s years of schooling	0.002* (− 0.000–0.003)	0.002* (− 0.000–0.003)	0.003*** (0.001–0.006)	0.002** (0.000–0.004)	0.000 (− 0.001–0.002)	0.002*** (0.001–0.003)
Age of the head	0.000 (− 0.000–0.001)	0.001*** (0.000–0.001)	0.000 (− 0.000–0.001)	0.000 (− 0.000–0.001)	0.000(− 0.001–0.001)	0.000 (− 0.000–0.001)
Female head	0.034* (− 0.001–0.069)	0.005 (− 0.043–0.054)	− 0.036* (− 0.079–0.007)	0.009 (− 0.031–0.048)	0.000 (− 0.032–0.032)	0.002 (− 0.025–0.030)
Household size	− 0.001 (− 0.004–0.001)	− 0.004** (− 0.008 to − 0.001)	− 0.003 (− 0.007–0.001)	− 0.004** (− 0.007 to − 0.001)	0.001 (− 0.003–0.004)	− 0.004*** (− 0.006 to − 0.002)
Standard of living index	0.004*** (0.002–0.005)	0.007*** (0.005–0.010)	0.005*** (0.003–0.007)	0.006*** (0.004–0.007)	− 0.006*** (− 0.007 to − 0.004)	0.009*** (0.007–0.011)
Distance to nearest facility	− 0.001 (− 0.004–0.001)	− 0.001 (− 0.003–0.002)	− 0.001 (− 0.005–0.002)	− 0.003** (−0.006 to − 0.000)	− 0.004*** (− 0.006 to − 0.002)	0.001 (− 0.001–0.004)
Observations	9455	9503	9426	9503	9503	9503

Reported coefficients are probit marginal effects. Regressions include religion and social group dummies, district fixed effects, delivery year fixed effects, and district-level pre-JGY health status interacted with delivery year dummies. Standard errors clustered at the district level. 95% confidence intervals in parentheses. ***p < 0.01, **p < 0.05, *p < 0.1

### Robustness checks

We check the robustness of our main findings using alternate methodology and alternate data. First, to further reduce potential bias in our samples from Gujarat and Maharashtra for the health information and health services utilization outcomes, we match the samples from DLHS-II and DLHS-III separately on observed characteristics using nearest neighbor propensity score matching (PSM). The observed characteristics are the same as those listed in Table 2. We then use only the matched sample from the two rounds, that is, observations that satisfy the common support assumption, to perform the difference-in-differences analysis. The results are consistent with our main findings as shown in Additional file 1: Tables S1–S3. Owing to relatively small sample size of PHCs and few observed characteristics on which PHCs from Gujarat and Maharashtra could be matched, PSM could not be performed for the health facilities outcomes.

Second, we re-run the health information and health services utilization using DLHS-II and the fourth round of the National Family Health Survey (NFHS) conducted in 2015–2016 [27]. DLHS-II sample from Gujarat and Maharashtra is our pre-JGY data and NFHS-IV is post-JGY data. We could not use NFHS-III conducted in 2005–2006 as pre-JGY data as the survey was conducted midway through JGY implementation, and we therefore cannot identify sufficient pre-JGY sample from Gujarat. DLHS is representative at the district level, while NFHS is representative at the state level. However, since we use JGY district level information only to identify the appropriate samples for analyses and otherwise define the JGY treatment variable at the state level, the difference in sample representativeness is not a concern. The sample sizes across DLHS-II and NFHS-IV for child immunization and maternal health services are very similar. However, the sample size for health information in NFHS-IV is much smaller. We could not perform the analysis for health facilities outcomes as NFHS does not contain a facilities survey. We use outcome and control variables in NFHS-IV that are comparable to DLHS-II as shown in Additional file 1: Table S4.

Our findings for health services utilization, that is, child immunization and maternal health services, are largely consistent in terms of direction of the effect and somewhat consistent in terms of statistical significance as shown in Additional file 1: Tables S6 and S7. As NFHS-IV was conducted nearly 8 years after the implementation of JGY across Gujarat, these findings suggest that effects of reliable electricity on health services utilization can sustain in the long term. However, we do not find any significant effects on health information received via television. A plausible explanation for this is that in recent years, even in rural India, health information is more likely to be accessed via mobile phones rather than television [28].

## Discussion

The overall picture that emerges from our analysis is that JGY implementation in Gujarat, which improved the reliability of electricity both in terms of hours of supply and voltage stability, had a significant positive effect on core components of health systems including health facilities, health information, and child and maternal health services utilization. The effect on health facilities (PHCs) is direct with improvements across most of the essential devices and equipment. Such supply-side improvements are crucial in raising the health status in developing countries, especially in rural areas. PHCs are essential providers within the network of government-funded health centers that provide free health services to rural residents. With the density of PHCs in India being low at approximately one PHC per 30,000 rural residents, maintaining essential devices and equipment becomes even more critical in providing high-quality health services without disruption [29]. In turn, proper maintenance and functioning of essential devices and equipment such as refrigerators, vaccine carriers, and operating table require reliable electricity [2, 4, 30]. Improvements in PHCs brought about by reliable electricity can therefore ensure continued access to health care and also facilitate high-quality health services provision.

With regards to health information, we find that the probability of accessing health information via television increases significantly with more reliable electricity. It has been previously found that electricity can increase exposure to television and internet and consequently to health information and education campaigns relayed via these electronic media [31–34]. As a large share of rural residents in India, especially women, are illiterate, it is much easier and effective for them to access information through television rather than newspapers or other print media. Reliable electricity can therefore increase overall health knowledge received by rural residents, particularly rural mothers, by increasing television viewing. Our survey data does not contain questions on health information accessed through the internet. However, it seems almost inevitable that with reliable electricity, greater penetration of affordable smart phones, and ease of charging phone batteries, access to health information via internet is likely to increase manifold [28]. Increased health information can in turn generate a positive feedback loop by increasing the demand for and utilization of health services.

On the demand side, health services utilization is pivotal to health systems and in actually achieving the health SDGs. We find that reliable electricity increases the probability of utilizing child immunization and maternal health services, among the most important health SDGs. Increase in accessing child immunization and maternal health services can result from positive spillovers between the different health system components. With well-equipped and well-functioning PHCs accessible within reasonable distance, mothers and pregnant women can easily avail health services [13, 35]. Indeed, our data shows a positive correlation between improved health facilities and health services utilization, and the correlation is stronger for health facilities in Gujarat (see Additional file 1: Figure S3a–l). Health information received through television and health workers can further persuade them to immunize their children and get regular check-ups during pregnancy [14, 31]. While we do not find any effect of reliable electricity on institutional delivery in PHCs, it can be expected to increase gradually owing to improved operational capacity of PHCs, particularly the availability of a functioning delivery table and operating table, coupled with support from other tiers of the rural health system, such as sub-centers and community health centers.

Specific to immunization, availability of cold storage facilities is crucial as both vaccine safety and potency are affected if vaccines are not transported and stored at recommended temperatures. It is also important that the vaccine cold chain at the lower levels of the health network (PHCs) links up with the higher order chain (vaccine suppliers). To be effective, it is recommended that specific cold chain equipment be made available and be properly maintained such as refrigerator/freezer, thermometer, cold box, ice-lined refrigerators, and vaccine carriers [36]. There is therefore a close link between proper maintenance of essential devices and equipment in PHCs and increased child immunization.

Besides interaction of different components within health systems, reliable electricity can also bring about changes in household time-use that can positively influence utilization. In particular, reliable electricity can result in a “time endowment” effect. This is because electrification makes households more efficient in labor-intensive activities and also effectively increases the length of the day [19, 37]. This time freed from labor-intensive activities or time gained due to lengthening of the day can potentially be allocated to accessing health services [38]. At the same time, health facilities can extend their hours of service provision per day owing to the availability of electricity [4].

Reliable electricity thus results in positive direct and indirect effects on core components of health systems. The strengths of our study are matching the administrative data on JGY implementation with DLHS survey data to identify the appropriate samples for analysis and applying the difference-in-differences framework to address potential bias in the implementation of JGY. Despite our application of rigorous statistical methods, our study still has methodological limitations. As JGY is not a randomized policy intervention, it is possible that we have not captured all unobserved confounding factors that could be simultaneously correlated with the JGY implementation and outcome variables.

## Conclusion

To our knowledge, this is the first study to provide rigorous empirical evidence on the effects of large-scale infrastructure improvements on health systems in a developing country. Our results indicate that reliable electricity can be an effective tool in improving core components of health systems such as improving the operational capacity of PHCs, improving the access to health information through electronic media, and boosting the utilization of child immunization and maternal health services.

Our research underscores the need for health policymakers to realize that in addition to targeting direct factors within the health systems such as health workforce and health financing, synergies between the health and infrastructure sectors need to be identified and promoted to effectively overcome non-monetary barriers such as quality of service, information, and time and to consequently achieve health goals. There are at least two further implications of our study. First, rural electrification or rural infrastructure improvements more broadly can reduce urban-rural health inequities. Second, besides immunization and maternal health services, reliable electricity can result in additional positive spillovers such as better tuberculosis and HIV/AIDS diagnostics, which can be explored in future research [4].

## Additional file

Additional file 1: **Figure S1.** (a) and (b). Pre- and post-JGY feeder system. **Figure S2.** JGY program implementation by district. **Figure S3.** Correlation between health facility improvement and health services utilization. **Table S1.** Effect of JGY implementation on health information: robustness check using difference-in-differences with matched samples. **Table S2.** Effect of JGY implementation on health services utilization (child immunization): robustness check using difference-in-differences with matched samples. **Table S3.** Effect of JGY implementation on health services utilization (maternal health): robustness check using difference-in-differences with matched samples. **Table S4.** Details of outcome variables, sample, and control variables included in the analysis: robustness check using NFHS data. **Table S5.** Effect of JGY implementation on health information: robustness check using NFHS data. **Table S6.** Effect of JGY implementation on health services utilization—child immunization: robustness check using NFHS data. **Table S7.** Effect of JGY implementation on health services utilization—maternal health services: robustness check using NFHS data. (DOCX 421 kb)

## Abbreviations

DLHS: District Level Household and Facility Survey
JGY: Jyotigram Yojana
NFHS: National Family Health Survey
PHC: Primary health center
