# Supplementary material for: Effect of reliable electricity on health facilities, health information, and child and maternal health services utilization: evidence from rural Gujarat, India
Source: J Health Popul Nutr. 2019 Feb 19;38:7. doi: 10.1186/s41043-019-0164-6 (PMC6381650; doi:10.1186/s41043-019-0164-6)
Supplement: Supplementary file 1 — Figure S1. (a) and (b). Pre- and post-JGY feeder system. Figure S2. JGY program implementation by district. Figure S3. Correlation between health facility improvement and health services utilization. Table S1. Effect of JGY implementation on health information: robustness check using difference-in-differences with matched samples. Table S2. Effect of JGY implementation on health services utilization (child immunization): robustness check using difference-in-differences with matched samples. Table S3. Effect of JGY implementation on health services utilization (maternal health): robustness check using difference-in-differences with matched samples. Table S4. Details of outcome variables, sample, and control variables included in the analysis: robustness check using NFHS data. Table S5. Effect of JGY implementation on health information: robustness check using NFHS data. Table S6. Effect of JGY implementation on health services utilization—child immunization: robustness check using NFHS data. Table S7. Effect of JGY implementation on health services utilization—maternal health services: robustness check using NFHS data. (DOCX 421 kb) [file 41043_2019_164_MOESM1_ESM.docx]

**Additional file 1**

Figures S1(a) and S1(b). Pre- and post-JGY feeder system

S1b: Feeder system after JGY

S1a: Feeder system before JGY

Source: Shah [1]

Reference:

1Shah T, Bhatt S, Shah RK, Talati J. Groundwater Governance Through Electricity Supply Management: Assessing an Innovative Intervention in Gujarat, Western India. Agricultural Water Management. 2008;95(11):1233-42.

Figure S2**.** JGY program implementation by district


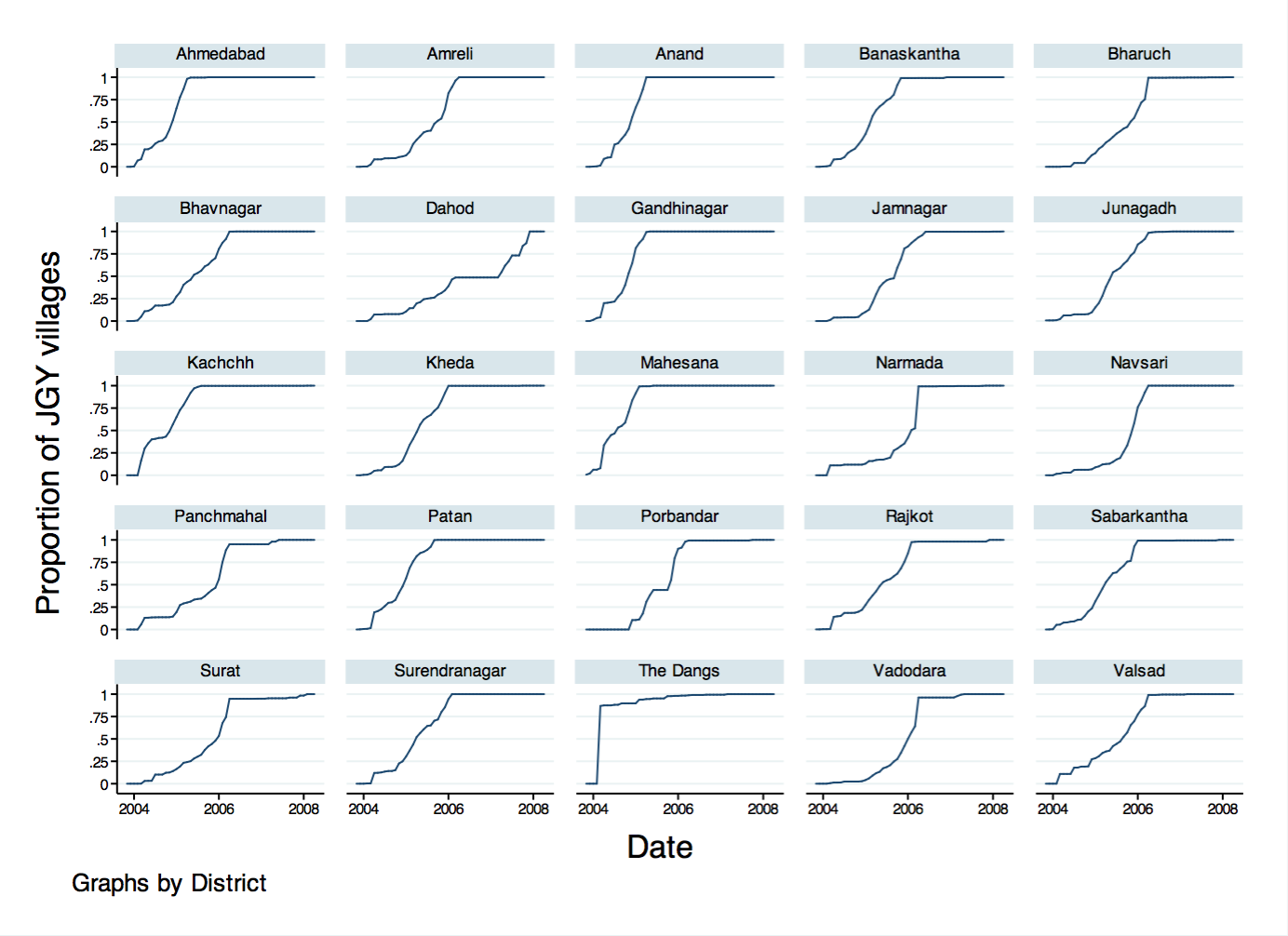


Source: Administrative data on JGY program implementation collected from electricity distribution companies in Gujarat by the authors.

Figure S3**.** Correlation between health facility improvement and health services utilization

We plot “health facility index” against the change in mean health services utilization (pre- and post-JGY) for both Gujarat and Maharashtra. Health facility index is a sum score of all available essential devices and equipment at a PHC as listed in Table 2 (under health facility outcome variables).

We observe that, overall, there is a positive correlation between health facility index and health services utilization. Upon further examination, we observe that the positive correlation is stronger for PHCs in Gujarat compared to those in Maharashtra as observations from Gujarat tend to be to the right (higher facility index score) and above the line for fitted values (stronger correlation). The exception is the graph for delivery in a private health facility for which we expected a negative correlation as improved PHC operational capacity would likely reduce reliance on private health services as they often entail out-of-pocket expenditure.

| (a) Correlation between health facility index and change in receiving first dose of DPT vaccine    $Y=0.0663+0.061*Change in Health Facility Index$  $R^{2}=0.032$ | (b) Correlation between health facility index and change in receiving all three doses of DPT vaccine  ****  $Y=-0.062+0.017*Change in Health Facility Index$  $R^{2}=0.001$ |
| --- | --- |
| (c) Correlation between health facility index and change in receiving first dose of polio vaccine  ****  $Y=0.143+0.082*Change in Health Facility Index$  $R^{2}=0.048$ | (d) Correlation between health facility index and change in receiving all three doses of polio vaccine  ****  $Y=0.004+0.042*Change in Health Facility Index$  $R^{2}=0.006$ |

| (e) Correlation between health facility index and change in receiving BCG vaccine  ****  $Y=0.119+0.041*Change in Health Facility Index$  $R^{2}=0.021$ | (f) Correlation between health facility index and change in receiving measles vaccine  ****  $Y=0.063+0.103*Change in Health Facility Index$  $R^{2}=0.024$ |
| --- | --- |
| (g) Correlation between health facility index and change in receiving at least one ANC check-up  **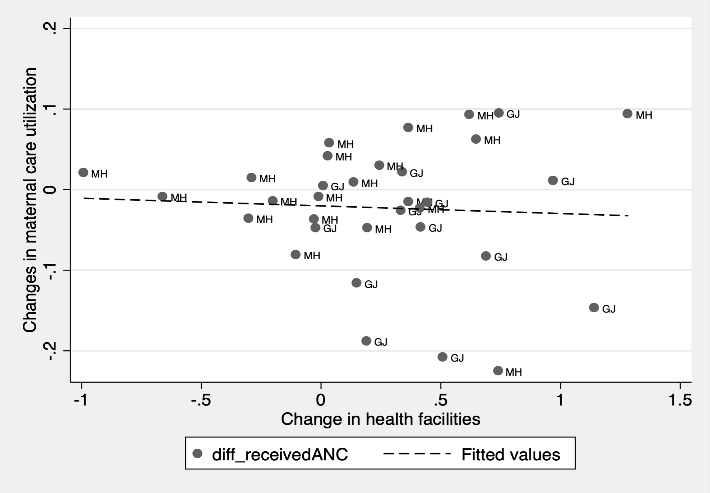**  $Y=-0.020-0.009*Change in Health Facility Index$  $R^{2}=0.003$ | (h) Correlation between health facility index and change in receiving at least three ANC check-ups  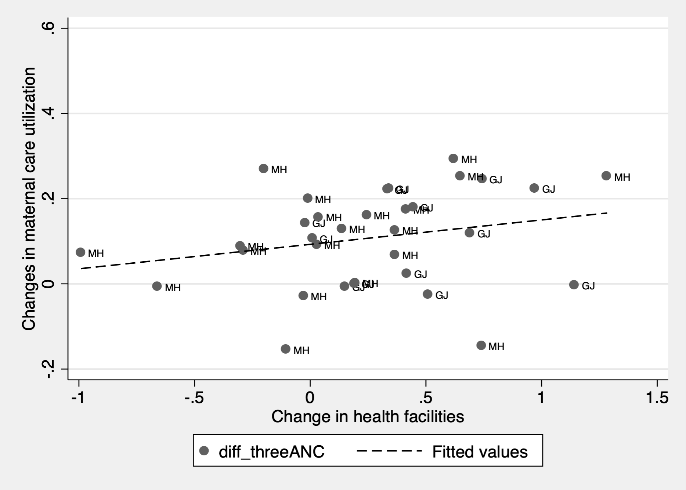  $Y=0.093+0.057*Change in Health Facility Index$  $R^{2}=0.056$ |
| (i) Correlation between health facility index and change in receiving ANC check-up in the first trimester  **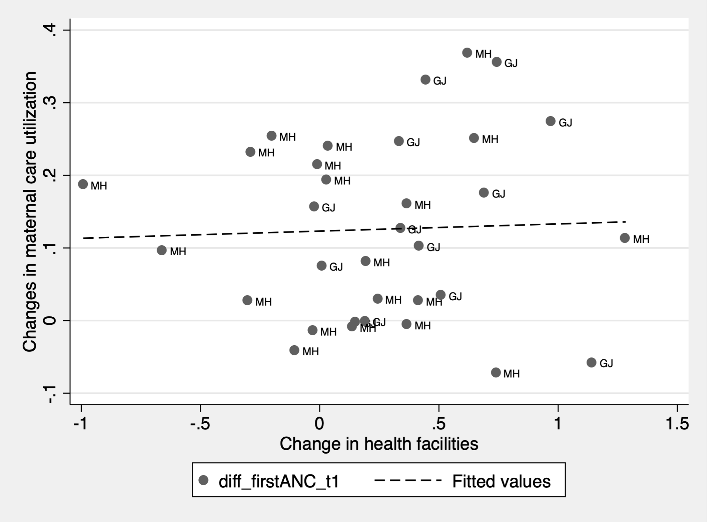**  $Y=0.123+0.010*Change in Health Facility Index$  $R^{2}=0.001$ | (j) Correlation between health facility index and change in delivering in a health facility  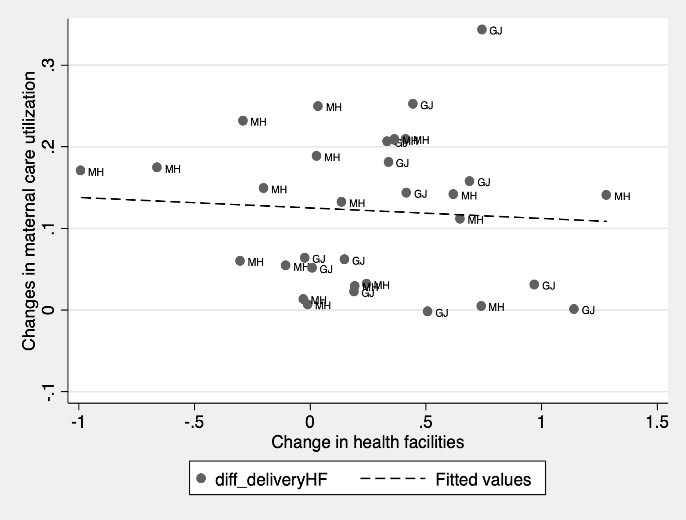  $Y=0.125-0.013*Change in Health Facility Index$  $R^{2}=0.005$ |

| (k) Correlation between health facility index and change in delivering in a public health facility  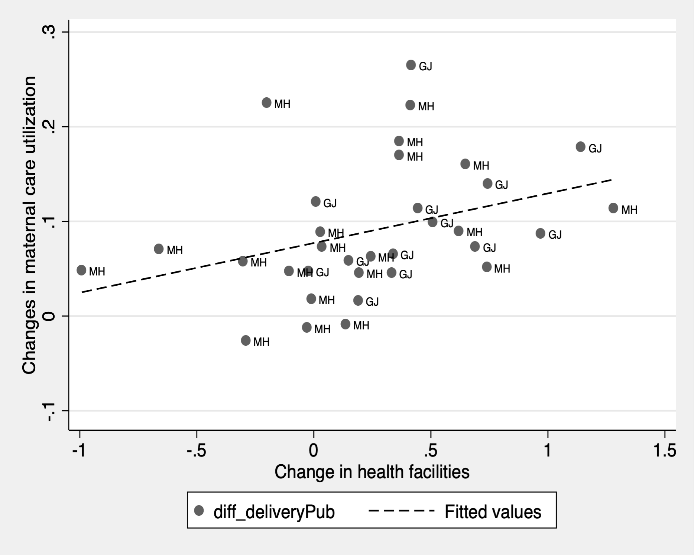  $Y=0.077+0.052*Change in Health Facility Index$  $R^{2}=0.126$ | (l) Correlation between health facility index and change in delivering in a private health facility  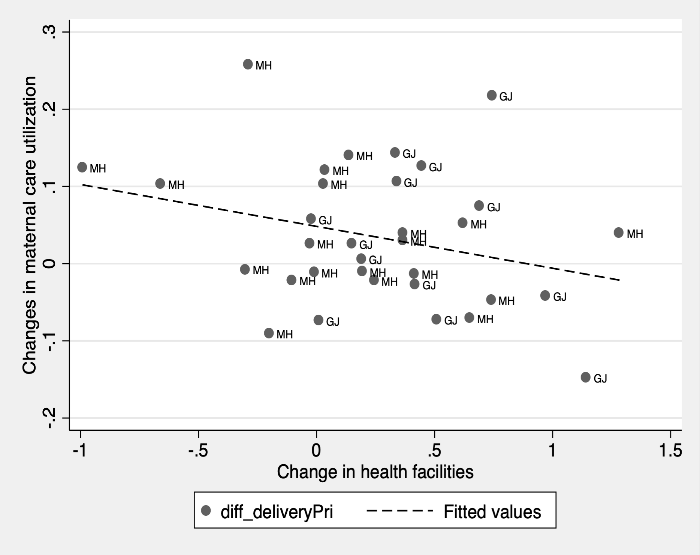  $Y=0.048-0.054*Change in Health Facility Index$  $R^{2}=0.081$ |
| --- | --- |

Table S1. Effect of JGY implementation on health information: Robustness check using difference-in-differences with matched samples

| **VARIABLES** | **(1)** | **(2)** | **(3)** |
| --- | --- | --- | --- |
|  | **Ever heard of HIV/AIDS** | **Heard from TV** | **Heard from Any Other Source** |
| Post*Gujarat (JGY program effect) | -0.001 | 0.098*** | -0.031 |
|  | (-0.046 – 0.044) | (0.038 - 0.158) | (-0.096 – 0.033) |
| Observations | 34,430 | 18,093 | 18,093 |

Notes: Reported coefficients are probit marginal effects from difference-in-differences analysis with matched samples. Matching was performed using nearest neighbour propensity score matching (PSM) on the following observed variables: age of the eligible woman, age squared, years of education of eligible woman, husband’s years of education, age of the household head, gender of the household head, religion and social group of the eligible woman, household standard of living index, and distance to nearest health facility. Only observations on the common support were used to perform the difference-in-differences analysis. Difference-in-differences regressions also include district fixed effects, interview year fixed effects, and district-level pre-JGY health status interacted with interview year dummies. Standard errors were bootstrapped with 1000 iterations. 95% confidence intervals in parentheses. *** p<0.01, ** p<0.05, * p<0.1.

Table S2. Effect of JGY implementation on health services utilization (child immunization): Robustness check using difference-in-differences with matched samples

| **VARIABLES** | **(1)** | **(2)** | **(3)** | **(4)** | **(5)** | **(6)** |
| --- | --- | --- | --- | --- | --- | --- |
|  | **DPT** | | **Polio** | | **BCG** | **Measles** |
|  | **First dose** | **All doses** | **First dose** | **All doses** |  |  |
| Post*Gujarat (JGY program effect) | 0.048** | 0.082** | 0.034** | 0.056* | 0.081*** | 0.160*** |
|  | (0.001 – 0.094) | (0.019 – 0.145) | (-0.004 - 0.071) | (-0.005 – 0.117) | (0.046 – 0.117) | (0.092 – 0.228) |
| Observations | 8,988 | 8,285 | 9,044 | 8,302 | 8,792 | 8,980 |

Notes: Reported coefficients are probit marginal effects from difference-in-differences analysis with matched samples. Matching was performed using nearest neighbour propensity score matching (PSM) on the following observed variables: Child’s age in months, mother’s age at birth, mother’s years of education, father’s years of education, birth order, dummy for multiple birth, age of the household head, gender of the household head, religion and social group of the child, household size, household standard of living index, and distance to nearest health facility. Only observations on the common support were used to perform the difference-in-differences analysis. Difference-in-differences regressions also include district fixed effects, birth year fixed effects, and district-level pre-JGY health status interacted with birth year dummies. Standard errors were bootstrapped with 1000 iterations. 95% confidence intervals in parentheses. *** p<0.01, ** p<0.05, * p<0.1.

Table S3. Effect of JGY implementation on health services utilization (maternal health): Robustness check using difference-in-differences with matched samples

| **VARIABLES** | **(1)** | **(2)** | **(3)** | **(4)** | **(5)** | **(6)** |
| --- | --- | --- | --- | --- | --- | --- |
|  | **At least one check-up** | **At least three check-ups** | **Check-up in the first trimester** | **Delivery in health facility** | **Delivery in public facility** | **Delivery in private facility** |
| Post*Gujarat (JGY program effect) | 0.013 | 0.045 | 0.079** | -0.023 | 0.053* | -0.054* |
|  | (-0.035 – 0.061) | (-0.023 – 0.114) | (0.010 – 0.149) | (-0.093 – 0.047) | (-0.006 – 0.112) | (-0.111 – 0.004) |
| Observations | 9,455 | 9,430 | 9,426 | 9,503 | 9,503 | 9,503 |

Notes: Reported coefficients are probit marginal effects from difference-in-differences analysis with matched samples. Matching was performed using nearest neighbour propensity score matching (PSM) on the following observed variables: eligible woman’s age at delivery, age squared, total number of births, total number of pregnancies, years of education of eligible woman, husband’s years of education, age of the household head, gender of the household head, religion and social group of the eligible woman, household size, household standard of living index, and distance to nearest health facility. Only observations on the common support were used to perform the difference-in-differences analysis. Difference-in-differences regressions also include district fixed effects, delivery year fixed effects, and district-level pre-JGY health status interacted with delivery year dummies. Standard errors were bootstrapped with 1000 iterations. 95% confidence intervals in parentheses. *** p<0.01, ** p<0.05, * p<0.1.

Table S4. Details of outcome variables, sample, and control variables included in the analysis: Robustness check using NFHS data

| **Outcome variables** | **Variable description** | **Unit of analysis** | **Sample** | **Control variables** |
| --- | --- | --- | --- | --- |
| **II] Health information** |  |  |  |  |
| 1. Ever heard of HIV/AIDS   If heard, heard from:   1. TV 2. Any other source | Dummy variable taking value 1 if response is “Yes”, 0 otherwise. | DLHS-2: Eligible woman  (women aged 18-45 that ever gave birth)  NFHS-IV: Eligible woman  (women aged 18-45 that ever gave birth) | Timing of JGY implementation is matched with timing of the household interviews in Gujarat. Sample includes eligible women interviewed in DLHS-II (pre-JGY) and NFHS-IV (post-JGY) from Gujarat and Maharashtra but excluding those residing in districts in Gujarat where JGY was already implemented in 2003-2004. | Age of the eligible woman, age squared, years of education of eligible woman, age of the household head, gender of the household head, religion and social group of the eligible woman, household standard of living index, district fixed effects, and interview year fixed effects.  In addition, district-level pre-JGY health status (proxied using under-five mortality rate in 2001) interacted with interview year dummies is included to control for unobserved differences across districts that may violate the parallel trend assumption. |
|  | | | | |
| **III] Health services utilization** |  |  |  |  |
| ***A) Child immunization services***   1. Received first DPT dose 2. Received all three DPT doses 3. Received first polio dose 4. Received all three polio doses 5. Received BCG vaccine 6. Received measles vaccine | Dummy variable taking value 1 if the child receives the specific immunization, 0 otherwise. | Child  (children between 0-30 months old at the time of survey) | Timing of JGY implementation is matched with timing of birth of a child such that only those children in Gujarat born before JGY implementation in DLHS-II and those born after JGY implementation in NFHS-IV are retained in the sample. All children from Maharashtra in DLHS-II and NFHS-IV are included. | Child’s age in months, mother’s age at birth, mother’s years of education, birth order, dummy for multiple birth, age of the household head, gender of the household head, religion and social group of the child, household size, household standard of living index, district fixed effects, and birth year fixed effects.  In addition, district-level pre-JGY health status (proxied using under-five mortality rate in 2001) interacted with birth year dummies is included to control for unobserved differences across districts that may violate the parallel trend assumption. |
| ***B) Maternal health services (antenatal care and institutional delivery)***   1. Received at least one ANC check-up 2. Received at least three ANC check-ups 3. Received ANC check-up in the first trimester 4. Delivery in a health facility 5. Delivery in a public health facility 6. Delivery in a private health facility | Dummy variable taking value 1 if the pregnant woman receives the specific ANC service, 0 otherwise. | Eligible woman  (women who gave live or still birth to a child in the past 2 years) | Timing of JGY implementation is matched with the timing of delivery such that only those eligible women in Gujarat who gave birth prior to JGY implementation in DLHS-II and those in NFHS-IV who gave birth after JGY implementation are retained in the sample. All eligible women from Maharashtra in DLHS-II and NFHS-IV are included. | Eligible woman’s age at delivery, age squared, total number of births, total number of pregnancies, years of education of eligible woman, age of the household head, gender of the household head, religion and social group of the eligible woman, household size, household standard of living index, district fixed effects, and delivery year fixed effects.  In addition, district-level pre-JGY health status (proxied using under-five mortality rate in 2001) interacted with delivery year dummies is included to control for unobserved differences across districts that may violate the parallel trend assumption. |

Notes:

1) We dropped husband’s years of education from the analysis as there are too many missing values for that variable in NFHS-IV.

2) We converted household standard of living to a discrete measure owing to data availability in NFHS-IV.

3) We changed the children’s age restriction to 0-30 months to make the DLHS-II and NFHS-IV samples consistent.

4) We could not include distance to health facility as a control variable as the distance questions in DLHS-II and NFHS-IV are not directly comparable.

Table S5. Effect of JGY implementation on health information: Robustness check using NFHS data

| **VARIABLES** | **(1)** | **(2)** | **(3)** |
| --- | --- | --- | --- |
|  | **Ever heard of HIV/AIDS** | **Heard from TV** | **Heard from Any Other Source** |
| Post*Gujarat (JGY program effect) | 0.041 | -0.100 | 0.117 |
|  | (-0.116 - 0.198) | (-0.251 - 0.051) | (-0.029 - 0.263) |
| Observations | 14,301 | 7,758 | 7,758 |

Notes: Reported coefficients are probit marginal effects from difference-in-differences analysis. Controls variables include: age of the eligible woman, age squared, years of education of eligible woman, age of the household head, gender of the household head, religion and social group of the eligible woman, household standard of living index, district fixed effects, interview year fixed effects, and district-level pre-JGY health status interacted with birth year dummies. Standard errors clustered at the district level. 95% confidence intervals in parentheses. *** p<0.01, ** p<0.05, * p<0.1.

Table S6. Effect of JGY implementation on health services utilization – child immunization: Robustness check using NFHS data

| **VARIABLES** | **(1)** | **(2)** | **(3)** | **(4)** | **(5)** | **(6)** |
| --- | --- | --- | --- | --- | --- | --- |
|  | **DPT** | | **Polio** | | **BCG** | **Measles** |
|  | **First dose** | **All doses** | **First dose** | **All doses** |  |  |
| Post*Gujarat (JGY program effect) | 0.074** | 0.033 | 0.058* | -0.015 | 0.015 | 0.010 |
|  | (0.009 - 0.139) | (-0.023 - 0.089) | (-0.011 - 0.126) | (-0.088 - 0.057) | (-0.035 - 0.065) | (-0.043 - 0.062) |
| Observations | 8,012 | 7,310 | 8,018 | 7,276 | 8,019 | 8,009 |

Notes: Reported coefficients are probit marginal effects from difference-in-differences analysis. Controls variables include: child’s age in months, mother’s age at birth, mother’s years of education, birth order, dummy for multiple birth, age of the household head, gender of the household head, religion and social group of the child, household size, household standard of living index, district fixed effects, birth year fixed effects, and district-level pre-JGY health status interacted with delivery year dummies. Standard errors clustered at the district level. 95% confidence intervals in parentheses. *** p<0.01, ** p<0.05, * p<0.1.

Table S7. Effect of JGY implementation on health services utilization – maternal health services: Robustness check using NFHS data

| **VARIABLES** | **(1)** | **(2)** | **(3)** | **(4)** | **(5)** | **(6)** |
| --- | --- | --- | --- | --- | --- | --- |
|  | **At least one check-up** | **At least three check-ups** | **Check-up in the first trimester** | **Delivery in health facility** | **Delivery in public facility** | **Delivery in private facility** |
| Post*Gujarat (JGY program effect) | 0.042 | 0.064* | 0.129*** | 0.020 | -0.066 | 0.053 |
|  | (-0.015 - 0.099) | (-0.001 - 0.130) | (0.063 - 0.196) | (-0.042 - 0.081) | (-0.162 - 0.031) | (-0.013 - 0.120) |
| Observations | 6,884 | 7,215 | 6,944 | 6,937 | 6,940 | 6,940 |

Notes: Reported coefficients are probit marginal effects from difference-in-differences analysis. Controls variables include: Eligible woman’s age at delivery, age squared, total number of births, total number of pregnancies, years of education of eligible woman, age of the household head, gender of the household head, religion and social group of the eligible woman, household size, household standard of living index, district fixed effects, delivery year fixed effects, and district-level pre-JGY health status interacted with interview year dummies. Standard errors clustered at the district level. 95% confidence intervals in parentheses. *** p<0.01, ** p<0.05, * p<0.1.
